# Microfluidic White Organic Light-Emitting Diode Based on Integrated Patterns of Greenish-Blue and Yellow Solvent-Free Liquid Emitters

**DOI:** 10.1038/srep14822

**Published:** 2015-10-06

**Authors:** Naofumi Kobayashi, Takashi Kasahara, Tomohiko Edura, Juro Oshima, Ryoichi Ishimatsu, Miho Tsuwaki, Toshihiko Imato, Shuichi Shoji, Jun Mizuno

**Affiliations:** 1 Faculty of Science and Engineering, Waseda University 3-4-1 Okubo, Shinjuku, Tokyo 169-8555, Japan; 2 Development Department, Shutech Corporation, Ltd., 1-7-2 Aioicho, Sakata-shi, Yamagata 998-0032, Japan; 3 Frontier Materials Research Department, Materials Research Laboratories, Nissan Chemical Industries, Ltd., 488-6 Suzumi-cho, Funabashi, Chiba 274-0052, Japan; 4 Department of Applied Chemistry, Graduate School of Engineering, Kyushu University, 744 Motooka, Nishi, Fukuoka 819-0395, Japan; 5 Research Organization for Nano & Life Innovation, Waseda University, 513 Wasedatsurumakicho, Shinjuku, Tokyo 162-0041, Japan

## Abstract

We demonstrated a novel microfluidic white organic light-emitting diode (microfluidic WOLED) based on integrated sub-100-μm-wide microchannels. Single-μm-thick SU-8-based microchannels, which were sandwiched between indium tin oxide (ITO) anode and cathode pairs, were fabricated by photolithography and heterogeneous bonding technologies. 1-Pyrenebutyric acid 2-ethylhexyl ester (PLQ) was used as a solvent-free greenish-blue liquid emitter, while 2,8-di-tert-butyl-5,11-bis(4-tert-butylphenyl)-6,12-diphenyltetracene (TBRb)-doped PLQ was applied as a yellow liquid emitter. In order to form the liquid white light-emitting layer, the greenish-blue and yellow liquid emitters were alternately injected into the integrated microchannels. The fabricated electro-microfluidic device successfully exhibited white electroluminescence (EL) emission via simultaneous greenish-blue and yellow emissions under an applied voltage of 100 V. A white emission with Commission Internationale de l’Declairage (CIE) color coordinates of (0.40, 0.42) was also obtained; the emission corresponds to warm-white light. The proposed device has potential applications in subpixels of liquid-based microdisplays and for lighting.

White organic light-emitting diodes (WOLEDs) have attracted much attentions for the next generation of red, green, blue, and white (RGBW)-based displays as well as lighting applications because of their advantages such as plane-emission, large-scale, light-weight, and flexibility^1^,^2^,^3^,^4^,^5^,^6^,^7^. In order to produce white light, a broad emission band that covers the visible light wavelength is required. In general, an additive mixture of two complementary colors (yellow and blue, or greenish-blue)^1^,^2^,^3^ or three primary colors (red, green and blue)^4^,^5^,^6^,^7^ has been widely applied to obtain a broad emission spectrum. The basic structures of WOLEDs consist of solid-state organic semiconductor layers sandwiched between anode and cathode pairs. Three types of the emitting layer structures that can produce the white-light emission have been reported: stacked multilayers^1^,^2^, blended-layers based on the host-guest system^3^,^4^,^5^, and striped-layers^6^,^7^. The subpixels of displays require high-resolution patterns that are less than hundreds of μm in width^8^, and several studies have been reported on the patterning methods of the solid-state organic light-emitting layers, including vacuum deposition using a shadow mask^8^ and ink-jet printing^6^,^7^.

Recently there has been increasing interest in solvent-free organic fluids such as liquid organic semiconductors (LOSs) for novel organic electronic devices^9^. Instead of the solid-state organic layers, in 2009, Xu and Adachi proposed a unique OLED that uses an LOS as the emitting layer (i.e., a liquid-OLED), and an electroluminescence (EL) emission from the liquid-emitting layer was demonstrated^10^. The LOSs remain in a liquid state at room temperature without requiring organic solvents. The first liquid-OLED was simply fabricated by sandwiching a single liquid emitter between two glass substrates with electrodes. Over the past several decades, microfluidic devices have been developed mainly for biochemical fields because of their potential advantages such as fluid control, reduced-consumption of samples, and shorter reaction times^11^,^12^. Several fluid experiments have been carried out using state-of-the-art microfluidic devices with sub-100-μm-wide microchannels^13^.

In our previous work, we proposed a microfluidic OLED that combined liquid-OLEDs with microfluidic technologies toward a new type of liquid-based display in 2013 14. In that work, an electro-microfluidic device, which consisted of a single-μm-thick and 1000- to 1500-μm-wide negative photoresist SU-8 microchannels, was fabricated on a glass substrate using microelectromechanical systems (MEMS) process and a heterogeneous bonding technique through the use of self-assembled monolayers (SAMs). 1-Pyrenebutyric acid 2-ethylhexyl ester (PLQ), which is one of the pyrene-based LOSs, was injected into microchannels sandwiched between transparent anode and cathode pairs without a vacuum process to form the liquid emitting layers. The microfluidic OLED with PLQ successfully exhibited greenish-blue EL emissions through recombination of the holes and electrons at the light-emitting pixels of the microchannels.

This simple liquid emitting layer formation of microfluidic OLED is promising for realizing large-area displays and lightings. In addition, LOS is expected to produce truly flexible displays because liquid materials can easily change their shape. However, it is difficult to obtain the flexible liquid emitting layer making the best use of the flowability of LOSs in the above-proposed liquid-OLEDs and microfluidic OLEDs. Therefore, we developed the novel methodology for fabricating large-area flexible electro-microfluidic structure for liquid-OLEDs, and EL emissions were obtained from the flexible microfluidic OLED without any leakages in a bending state^15^.

Although LOSs are promising materials for novel liquid-based organic electronic devices, there are few materials available for the liquid emitting layers. Thus, the development of new LOS-based emitting layers is a challenge for realizing multicolor microfluidic OLED displays. Recently we proposed the use of PLQ not only as a greenish-blue emitter but also as a liquid host, and demonstrated on-demand multicolor microfluidic OLEDs using PLQ doped with the fluorescent guest emitters^16^. We also revealed the refreshable luminance characteristics of the microfluidic OLED via reinjection of fresh liquid emitters into the light-emitting pixels, indicating that deteriorated liquids were simply replaced with new liquids using microfluidic technologies. The microfluidic OLEDs have several unique properties such as flexible characteristics, on-demand multicolor emission on a single chip, and refreshable luminance feature, which are difficult to be realized with the conventional solid-state OLEDs. Further development of the microfluidic OLED having high-resolution LOS patterns is an important step toward functional liquid-based display and lighting applications.

In this study, we propose a microfluidic WOLED that can be used as the subpixels of the display, as shown in Fig. 1. Integrated sub-100-μm-wide microchannels sandwiched between two electrodes are formed in the microfluidic WOLED. Greenish-blue and yellow liquid emitters are alternately injected into the microchannels, and white-light emission can be produced by the simultaneous greenish-blue and yellow emissions.

## Experimental

**Figure 2:**
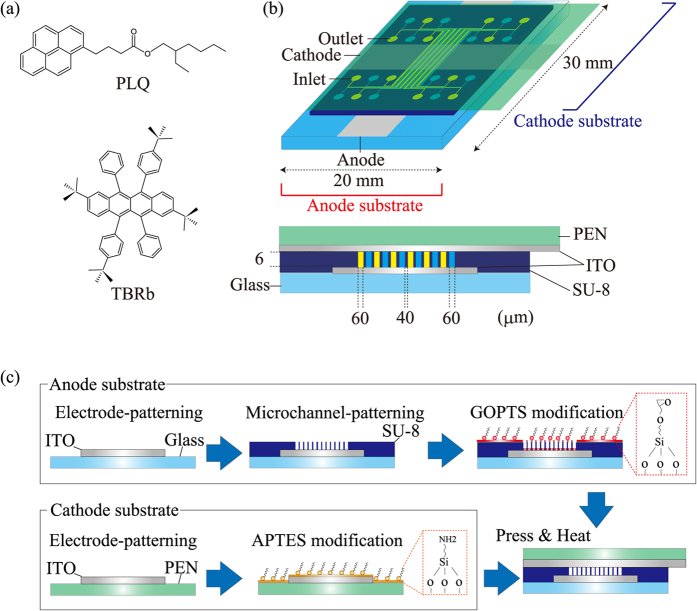
(**a**) Molecular structure of the employed materials. PLQ was used as the greenish-blue emitter and the host material. TBRb was used as the yellow fluorescent guest emitters. (**b**) Design of the microfluidic WOLED. Twelve SU-8 microchannels were sandwiched between the anode substrate and the cathode substrate. (**c**) Fabrication process of the microfluidic WOLED. Anode and cathode electrodes were patterned; subsequently, microchannels were fabricated by photolithography. Finally, two substrates were bonded with APTES- and GOPTS-SAMs.

Molecular structures of the employed materials are shown in Fig. 2(a). PLQ (Nissan Chemical Industries, Ltd.) was used as both the greenish-blue liquid emitter^14^ and the liquid host^15^. 2,8-Di-tert-butyl-5,11-bis(4-tert-butylphenyl)-6,12-diphenyltetracene (TBRb) (Luminescence Technology Co.) was used as a yellow fluorescent guest dopant^17^,^18^,^19^, and first doped into the host PLQ in accordance with the color tuning method reported in our previous works^15^. TBRb is promising as a highly efficient yellow fluorescent dopant because of its attractive properties such as bipolar characteristics^17^,^18^,^19^. TBRb was dissolved into CH_2_Cl_2_ and subsequently mixed with PLQ in a beaker. Finally, CH_2_Cl_2_ was evaporated by heating on a hotplate at 80°C in a vacuum chamber. In this study, we prepared 2wt% TBRb-doped PLQ. 0.25wt% tributylmethylphosphonium bis (trifluoromethanesulfonyl) imide (TMP-TFSI) (Tokyo Chemical Industry Co., Ltd.) was applied as the electrolyte and doped into the liquid emitters to enhance carrier injection^14^,^15^,^16^.

The design of the microfluidic WOLED is shown in Fig. 2(b). An indium tin oxide (ITO)-coated glass substrate was used as an anode substrate, while an ITO-coated polyethylene naphthalate (PEN) film substrate was used as a cathode substrate. Twelve SU-8 microchannels were sandwiched between a 3-glycidodyloxypropyltriethoxysilane (GOPTS)-modified ITO anode and aminopropyltriethoxysilane (APTES)-modified ITO cathode. The epoxy-terminated SAM of GOPTS and the amine-terminated SAM of APTES were used for the heterogeneous bonding of the anode and cathode substrates^20^,^21^. In order to meet the patterns with less than hundreds of μm that are required for subpixels of the display^8^, the microchannel widths were designed to be 60 μm, and the distance between the microchannels was 40 μm. The microchannel thickness was set to 6 μm in accordance with our previous work^14^. The emitting area is 1.44 mm^2^ (60 μm × 2000 μm × 12). PLQ and TBRb-doped PLQ were alternately injected into the microchannels to form the integrated greenish-blue and yellow patterns.

The fabrication process of the microfluidic WOLED is shown in Fig. 2(c). According to the fabrication process of the microfluidic OLED reported in our previous works^14^,^15^, the electrode patterns were formed on the anode and cathode substrates by wet etching with aqua regia; subsequently, the SU-8 3005 (MicroChem Co.) microchannels were fabricated on the anode substrate by photolithography. The surfaces of the anode and cathode substrates were modified with GOPTS- and APTES-SAMs, respectively, after vacuum ultraviolet/ozone (VUV/O_3_) pretreatments using a 172-nm excimer lamp (USHIO Inc., UER20-172)^14^. Finally, two substrates were bonded under a contact pressure of 1.5 MPa at 140 °C for 5 min using bonding equipment (SUSS MicroTech AG., SB6e). Since our previous microfluidic OLED had shallow microchannels with an aspect ratio (microchannel thickness/width) of 0.004 to 0.006, GOPTS-SAM was formed only on the SU-8 layer of the anode substrate to prevent conjugations between the ITO anode and cathode during the bonding process^14^. Here, both the SU-8 microchannels and ITO anodes were modified with GOPTS-SAM because the aspect ratio of the microfluidic WOLED consisting of 60-μm-wide and 6-μm-thick microchannels is two orders higher than that of the previous device.

The absorption spectrum of TBRb dissolved in dichloromethane (CH_2_Cl_2_) was obtained with an ultraviolet-visible (UV-Vis) spectrophotometer V-550 (JASCO). Photoluminescence quantum yield (PLQY) of PLQ and TBRb dissolved in acetonitrile (CH_3_CN) and CH_2_Cl_2_, respectively, was measured with an external quantum efficiency measurement system C9920-12 (Hamamatsu Photonics). Current density-voltage-luminance (*J-V-L*) characteristics were measured by using a DC power supply 2400 source meter (Keithley) and luminance meter LS-1100 (Konica-Minolta). Emission spectra and Commission Internationale de l,èclairage (CIE) coordinates were measured with spectrometer USB4000FL (Ocean Optics). The values of the CIE coordinates were expressed with ColorAC (Vector). Electroluminescent characteristics of PLQ and TBRb-doped PLQ were measured with the 6-μm-thick and 1000-μm-wide electro-microfluidic device, which was fabricated with the same process as that of the microfluidic WOLED.

## Results and Discussion

**Figure 3:**
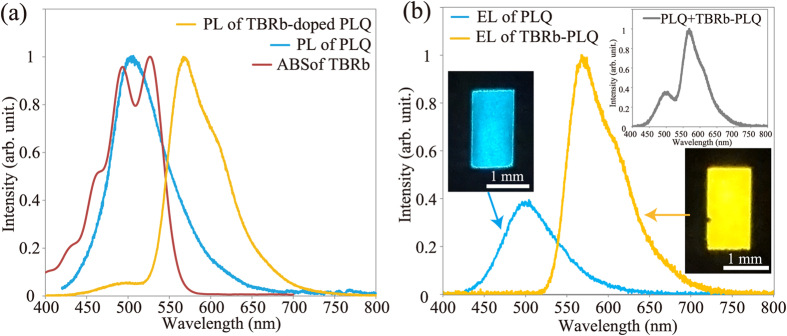
(**a**) PL spectrum of PLQ and 2wt% TBRb-doped PLQ, and absorption spectrum of 33.3 μM TBRb. (**b**) EL spectra of PLQ and 2wt% TBRb-doped PLQ with the microfluidic OLED. Inset: EL emissions of PLQ and TBRb-doped PLQ, and the sum of EL spectra of PLQ and 2wt% TBRb-doped PLQ under an applied voltage of 100 V.

**Figure 4:**
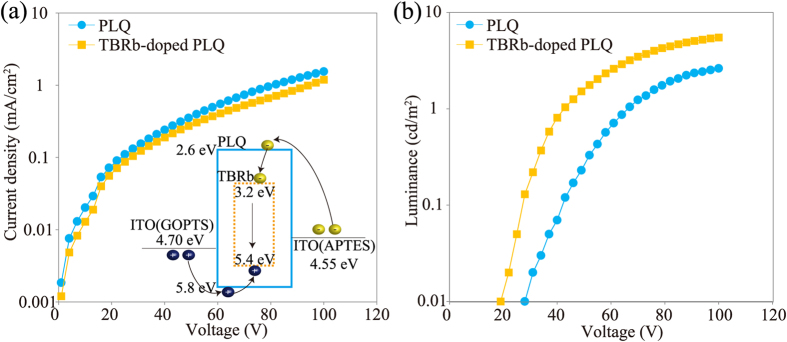
(**a**) *J-V* and (**b**) *L-V* characteristics of 6-μm-thick microfluidic OLED with PLQ and TBRb-doped PLQ, respectively. Inset: Energy diagram of TBRb-doped PLQ.

Figure 3(a) shows the absorption spectrum of 33.3 μM TBRb and the PL spectra of PLQ and TBRb-doped PLQ. An excitation wavelength of 365 nm was used for the PL measurements because the host PLQ has a strong UV absorption wavelength at less than 377 nm^15^,^16^,^22^. TBRb showed an absorption spectrum ranging from 400 nm to 570 nm. The PL emission of PLQ has a peak wavelength of 500 nm, and its spectrum overlaps with the absorption spectrum of TBRb. Although the host PLQ was selectively excited, TBRb-doped PLQ exhibited a peak PL wavelength of 560 nm. This result suggests that the Förster resonance energy transfer (FRET), which only allows for singlet-singlet energy transfer^23^, occurred from PLQ to TBRb. Figure 3(b) shows the EL spectra and photographic images of EL emissions. Greenish-blue and yellow EL emissions were confirmed from the 1000-μm-wide electro-microfluidic device with PLQ and TBRb-doped PLQ, respectively. In the case of PLQ, similar to the PL spectrum [Fig. 3(a)], an emission maximum at 500 nm and broad emission ranging from 420 nm to 650 nm were observed. The lowest unoccupied molecular orbital (LUMO) level and the highest occupied molecular orbital (HOMO) level of PLQ are 2.6 eV and 5.8 eV, respectively^15^,^22^. The work-function values of the GOPTS-modified ITO and APTES-modified ITO are 4.70 eV and 4.55 eV, respectively^15^. The EL emission confirms that the holes and electrons were injected from the GOPTS-modified ITO anode and APTES-modified ITO cathode, respectively, and then the excitons of PLQ were produced by the recombination of the holes and electrons. The electro-microfluidic device with TBRb-doped PLQ showed a broad emission ranging from 520 nm to 750 nm. The obtained EL emission maximum of TBRb-doped PLQ is identical to the PL emission maximum [Fig. 3(a)]. Although the emission attributed to the host PLQ was observed at approximately 500 nm in the PL spectrum, this emission was not clearly observed in the EL spectrum. The LUMO level and HOMO level of TBRb are reported to be located at 3.2 eV and 5.4 eV, respectively^17^,^18^. It was found that the HOMO-LUMO level of TBRb is within the HOMO-LUMO gap of the host PLQ. Therefore, the obtained EL spectrum suggests that the excitons of TBRb were generated not only by the FRET from the host to the guest but also by direct recombination of the electrons and holes which were trapped in the guest emitters^24^,^25^. From the EL spectrum measurements, TBRb-doped PLQ was found to be useful for the yellow liquid emitter. In addition, the sum of the EL spectra of PLQ and TBRb-doped PLQ is shown in the inset of Fig. 4(b). White EL emission, which has a broad spectrum ranging from 420 nm to 750 nm, would be produced by the simultaneous EL emissions of PLQ and TBRb-doped PLQ from the integrated microchannels. It was also found that the emission intensity of TBRb-doped PLQ is higher than that of PLQ at the same driving voltage of 100 V. PLQY of 10 μM PLQ and 18 μM TBRb were measured to be 69% and 74%, respectively. This result indicates that the enhancement in TBRb-doped system can be explained by the PLQY values. Thus, PLQ was found to be useful as the liquid host for a highly fluorescent yellow dopant of TBRb. From these results, the generation of warm-white light can be expected to be obtained by the proposed microfluidic WOLED having the same size of PLQ and TBRb-doped PLQ patterns.

*J-V* and *L-V* characteristics of the 1000-μm-wide electro-microfluidic devices are shown in Fig. 4(a,b), respectively. Similar *J-V* curves were confirmed from the devices with PLQ and TBRb-doped PLQ. This suggests that there are no significant differences between the bulk resistances of PLQ and TBRb-doped PLQ. It should be mentioned that in comparison with PLQ, a slight decrease in the current density was observed from the device with TBRb-doped PLQ. This decrease indicates that TBRb acts as a trapping molecule because the HOMO-LUMO gap of TBRb is within that of the host PLQ, as shown in the inset of Fig. 4(a)^26^. In this study, the emitting layers of the proposed microfluidic WOLED are designed to be formed by sandwiching PLQ and TBRb-doped PLQ between the common GOPTS-modified ITO anode and APTES-modified ITO cathode [see also Fig. 2(a)]. Thus, *J-V* measurements using the 1000-μm-wide electro-microfluidic devices revealed that both PLQ and TBRb-doped PLQ can be controlled by the same driving voltage for operating the microfluidic WOLED. As shown in Fig. 4(b), the electro-microfluidic device also exhibited stable luminescent characteristics, and the luminance values of the device with PLQ and TBRb-doped PLQ were 2.63 cd/m^2^ and 5.51 cd/m^2^ at 100 V, respectively. In addition, the device with TBRb-doped PLQ showed a lower turn-on voltage, which was defined at a luminance of above 0.01 cd/m^2^, than that with PLQ.

**Figure 5:**
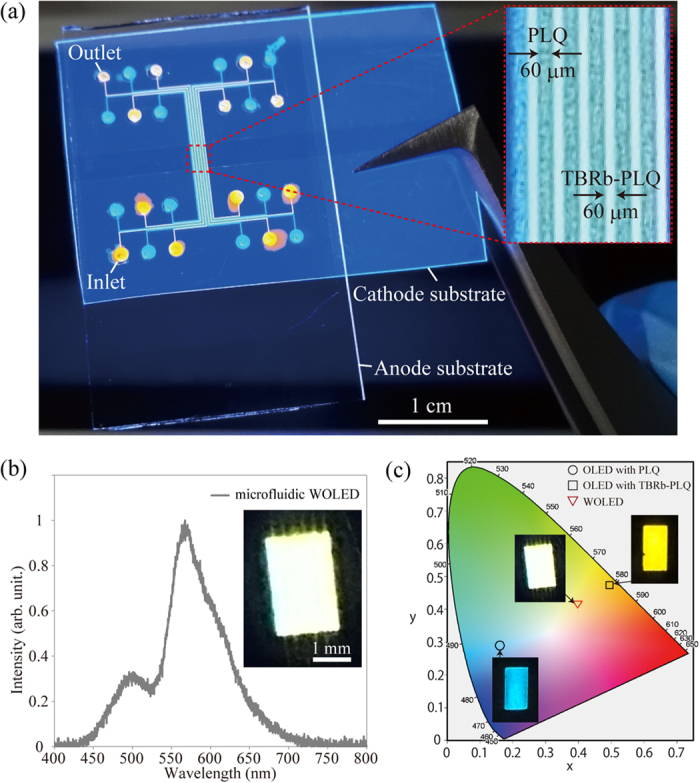
(**a**) Photographic image of the microfluidic WOLED under a 365-nm UV irradiation. (**b**) EL spectrum of the microfluidic WOLED under an applied voltage of 100 V. (**c**) CIE coordinates of the microfluidic OLEDs with PLQ and TBRb-doped PLQ, and the microfluidic WOLED.

Figure 5(a) shows a photographic image of the fabricated microfluidic WOLED. The liquid light-emitting layers were simply formed by alternately injecting the greenish-blue and yellow liquid emitters from the inlets into the 60-μm-wide microchannels. The liquid emitters were excited with a 365-nm UV lamp, which was introduced through the glass substrate of the microfluidic WOLED. The PLQ and TBRb-doped PLQ patterns were confirmed in the fabricated device without leakage at the bonded interface between the anode and cathode substrates. Therefore, the proposed fabrication methodology for the microfluidic WOLED is effective for realizing the integrated sub-100-μm wide liquid-emitting layers on a single device. The photographic image shown in Fig. 5(b) is the microfluidic WOLED under an applied voltage of 100 V. It can be clearly seen that white EL emission was successfully produced by the microchannels sandwiched between the GOPTS-modified ITO anode and APTES-modified ITO cathode. As shown in Fig. 5(b), the obtained spectrum was found to be composed of a greenish-blue emission band from PLQ and a yellow emission band from TBRb-doped PLQ. As a result, the microfluidic WOLED having the integrated PLQ and TBRb-doped PLQ patterns exhibited a broad emission band that covers the visible-light wavelength ranging from 420 nm to 750 nm Furthermore, the obtained spectrum shows that the yellow component was higher than the greenish-blue one at the applied voltage of 100 V; this is similar to EL spectra from the 1000-μm-wide electro-microfluidic device [see also Fig. 3(b)]. Thus, the excitons of greenish-blue and yellow emitters were successfully produced from the integrated 60-μm-wide microchannels as well as the 1000-μm-wide microchannels. CIE coordinates of the greenish-blue, yellow, and white-light emission under the applied voltage of 100 V are shown in Fig. 5(c). The obtained CIE coordinates of the 1000-μm-wide electro-microfluidic devices with PLQ and TBRb-doped PLQ at 100 V were (0.16, 0.26) and (0.49, 0.47), respectively. The microfluidic WOLED through the simultaneous greenish-blue and yellow emissions showed the CIE coordinates of (0.40, 0.42) at 100 V, which is within the white region and nearly corresponds to warm-white^27^. White balance of our microfluidic WOLED can be simply tuned by varying microchannel-width ratios for greenish-blue and yellow liquid emitters (see Fig. S1). This color-tunable characteristic with microfluidic technologies is an innovative property.

From the above results, the prototype microfluidic WOLED is useful in producing white EL emission from greenish-blue and yellow liquid emitters. We believe that the proposed microfluidic WOLED is expected to have potentials for applications in the liquid-based pixels of RGBW displays as well as in lighting. Moreover, the electro-microfluidic device having the 60-μm-wide microchannels would be expected to produce not only white emission consisting of greenish-blue and yellow components but also various color emissions from numerous combinations of different EL spectra. In our previous work, green and red EL emissions were realized using microfluidic OLEDs with 5,12-diphenyltetracene (DPT)- and tetraphenyldibenzoperiflanthene (DBP)-doped PLQ emitting layers, respectively^15^. For example, purple emission can be generated by the simultaneous emissions of PLQ and DBP-doped PLQ (see Fig. S2).

Although in comparison with state-of-the-art solid-state WOLED^2^,^27^, the characteristics of our microfluidic WOLED are still in a research stage, the device performance of the microfluidic WOLED is expected to be further improved by optimized device structures. For example, in the liquid-OLEDs sandwiched between two glass substrates, carrier injection has been reported to be improved by the introduction of additional layers^28^. In the case of a first liquid OLED reported by Xu and Adachi, cesium carbonate (Cs_2_CO_3_) was formed on an ITO cathode as an electron injection layer, while poly(3,4-ethylene dioxythiophene):poly(styrene sulfonate) (PEDOT:PSS) was formed on an ITO anode as a hole injection layer^10^. Hirata *et al.* introduced titanium dioxide (TiO_2_) on an ITO cathode as a hole-blocking layer^28^. The functional layer generates the electric dipole layers at the interfaces between the liquid emitting layers and electrodes, and reduces carrier injection barrier from the electrodes into the emitting layers. The driving voltage is also expected to be reduced with decreasing the microchannel depth because bulk resistance of the emitting layer depends on the emitter thickness^16^. Therefore, we plan to develop the microfluidic OLEDs with this kind of functional layers in the future. As mentioned above, our microfluidic OLED technology will be a highly promising technology for future unique light-emitting applications.

In conclusion, we proposed a microfluidic WOLED having sub-100-μm-wide microchannels with greenish-blue and yellow solvent-free liquid emitters. The guest dopant TBRb was doped into the host PLQ in order to obtain the yellow liquid emitter. The microfluidic WOLED was successfully fabricated by photolithography and heterogeneous bonding, and white EL emission was obtained under an applied voltage of 100 V. The demonstrated EL spectrum was covered by a broad emission band ranging from 420 nm to 750 nm. The CIE coordinates of (0.40, 0.42) were observed from the obtained white emission, which corresponds to warm-white light. Therefore, the prototype WOLED is expected to be useful for subpixels of liquid-based microdisplays and for lighting.

## Additional Information

**How to cite this article**: Kobayashi, N. *et al.* Microfluidic White Organic Light-Emitting Diode Based on Integrated Patterns of Greenish-Blue and Yellow Solvent-Free Liquid Emitters. *Sci. Rep.* **5**, 14822; doi: 10.1038/srep14822 (2015).

## Supplementary Material

Supplementary Information

## Figures and Tables

**Figure 1:**
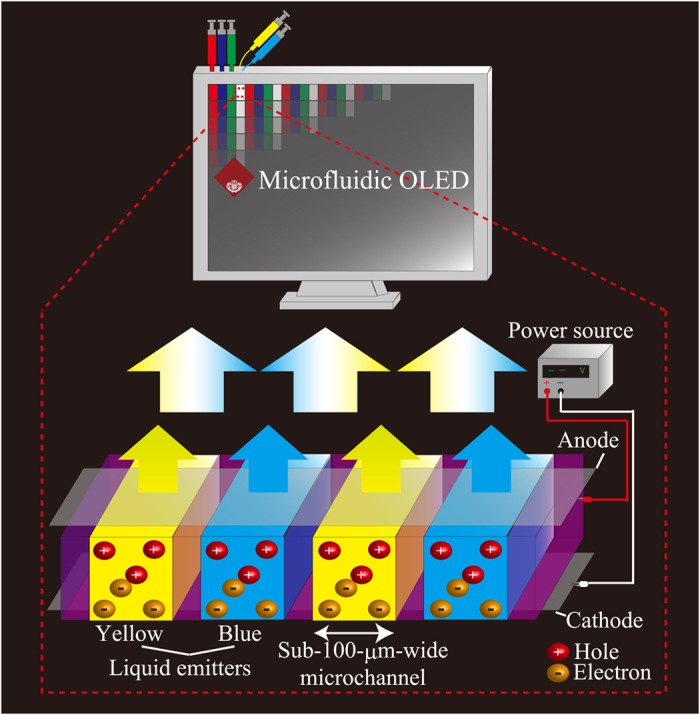
Concept of the microfluidic WOLED. Greenish-blue and yellow liquid emitters are alternately injected into the integrated microchannels, and white-light emission can be generated by the simultaneous emissions of the two different color emitters.
