# Supplementary material for: Microfluidic White Organic Light-Emitting Diode Based on Integrated Patterns of Greenish-Blue and Yellow Solvent-Free Liquid Emitters
Source: Sci Rep. 2015 Oct 6;5:14822. doi: 10.1038/srep14822 (PMC4594091; doi:10.1038/srep14822)
Supplement: Supplementary Information [file srep14822-s1.pdf]

## Supplementary Information:

# Microfluidic White Organic Light-Emitting Diode Based on Integrated Patterns of Greenish-Blue and Yellow Solvent-Free Liquid Emitters

Naofumi Kobayashi[1*], Takashi Kasahara[1*], Tomohiko Edura[2], Juro Oshima[3], Ryoichi Ishimatsu[4], Miho Tsuwaki[1], Toshihiko Imato[4], Shuichi Shoji[1], Jun Mizuno[5†]

[1]Faculty of Science and Engineering, Waseda University, 3-4-1, Okubo, Shinjuku, Tokyo 169-8555, Japan

[2]Development Department, Shutech Corporation, Ltd., 1-7-2 Aioicho, Sakata-shi, Yamagata, 998-0032 Japan

[3]Frontier Materials Research Department, Materials Research Laboratories, Nissan Chemical Industries, Ltd., 488-6 Suzumi-cho, Funabashi, Chiba 274-0052, Japan

[4]Department of Applied Chemistry, Graduate School of Engineering, Kyushu University, 744 Motooka, Nishi, Fukuoka 819-0395, Japan

[5]Research Organization for Nano & Life Innovation, Waseda University, 513 Wasedatsurumakicho, Shinjuku, Tokyo 162-0041, Japan

*These authors contributed equally to this article

†Corresponding author

## Effect of the microchannel widths for a yellow liquid emitter on EL spectra of microfluidic white organic light-emitting diodes

We investigated the effects of the microchannel widths for TBRb-doped PLQ on the white EL emissions of the microfluidic WOLED. Three types of the electro-microfluidic devices (Devices 1, 2, and 3) were fabricated by photolithography and heterogeneous bonding through the use of APTES- and GOPTS-SAMs, as shown in Table S1. All devices have the same microchannel width for PLQ. A greenish-blue liquid emitter PLQ and a yellow liquid emitter 2wt% TBRb-doped PLQ were alternately injected from the inlets into the 6-μm-thick microchannels sandwiched between GOPTS-modified ITO anode and APTES-modified ITO cathode.

Table S1 Microchannel widths of the yellow and greenish-blue liquid emitters in the electro-microfluidic devices.

| Sample | Yellow microchannel width (μm) | Greenish-blue microchannel width (μm) |
|---|---|---|
| Device 1 | 60 | 60 |
| Device 2 | 40 | 60 |
| Device 3 | 30 | 60 |

Figure S1 shows electroluminescence (EL) spectra from the devices 1, 2, and 3 at an applied voltage of 100 V. It was found that yellow components increased with increasing the microchannel widths for TBRb-doped PLQ. This suggests that our microfluidic WOLED can simply produce several white emissions by varying the channel-width ratios between PLQ and TBRb-doped PLQ.

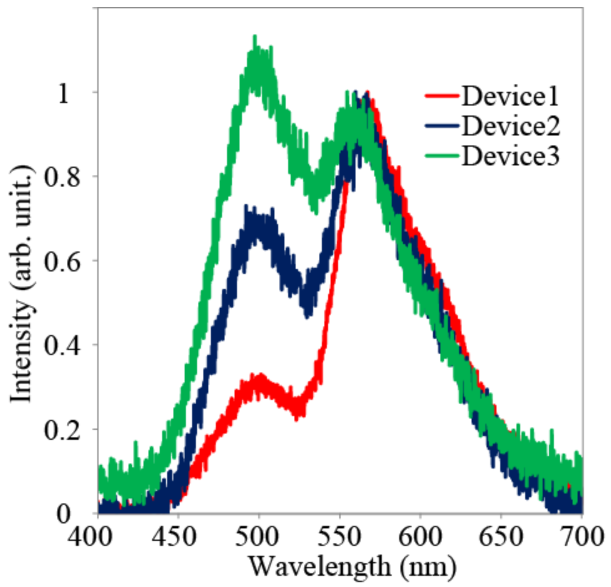


Figure S1 EL spectra of the microfluidic WOLED device varying the channel-width ratio between PLQ and TBRb-doped PLQ under an applied voltage of 100 V.

**Various color electroluminescence emission of the integrated microfluidic organic light-emitting diode**

We evaluated the electro-microfluidic device, which consists of the integrated 60-μm-wide microchannels, with combination of greenish-blue and red liquid emitters. Tetraphenyldibenzoperiflanthene (DBP)-doped PLQ were prepared as the red liquid emitter in accordance with the color tuning method reported in our previous works [S1]. PLQ and 0.4wt% DBP-doped PLQ were alternately injected from the inlets into the 60-μm-wide microchannels. Figure S2 shows a photograph of EL emission from the fabricated integrated microfluidic OLED with greenish-blue and red liquid emitters at an applied voltage of 70 V. It can be seen that purple emission was produced, indicating that the greenish-blue and red emissions were simultaneously generated at the emitting area of the electro-microfluidic device. We expect that our integrated electro-microfluidic device is also effective for generating full-color EL emissions via numerous combinations of liquid emitters.

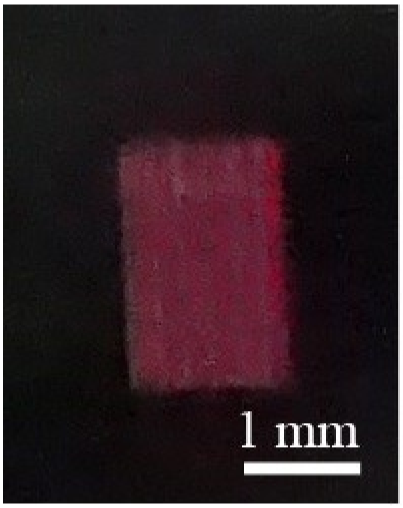


Figure S2 Purple EL emission from the fabricated microfluidic OLED device with integrated PLQ and 0.4wt% DBP-doped PLQ patterns under an applied voltage of 70V.

## Reference

[S1] Kasahara, T. et al. Multi-color microfluidic organic light-emitting diodes based on on-demand emitting layers of pyrene-based liquid organic semiconductors with fluorescent guest dopants. *Sens. Actuators. B. Chem.* **207**, 481-489 (2015).
